# Evaluation of Staging Systems for Cancer of the Nasal Vestibule

**DOI:** 10.3390/cancers15113028

**Published:** 2023-06-01

**Authors:** Willem Frederik Julius Scheurleer, Luca Tagliaferri, Johannes A. Rijken, Claudia Crescio, Davide Rizzo, Gian Carlo Mattiucci, Frank A. Pameijer, Remco de Bree, Bruno Fionda, Mischa de Ridder, Francesco Bussu

**Affiliations:** 1 Department of Head and Neck Surgical Oncology, University Medical Center Utrecht, Heidelberglaan 100, 3584 CX Utrecht, The Netherlands; 2 UOC Radioterapia Oncologica, Dipartimento di Diagnostica per Immagini, Radioterapia Oncologica ed Ematologia, Fondazione Policlinico Universitario ‘‘A. Gemelli’’ IRCCS, Largo Agostino Gemelli, 8, 00168 Rome, Italy; 3 Otolaryngology Division, Azienda Ospedaliero Universitaria Sassari, Viale S. Pietro 43, 07100 Sassari, Italy; 4 Department of Medicine, Surgery and Pharmacy, University of Sassari, Viale S. Pietro 43, 07100 Sassari, Italy; 5 Department of Radiation Oncology, Mater Olbia Hospital, SS 125 Orientale Sarda, 07026 Olbia, Italy; 6 Department of Radiology, University Medical Center Utrecht, Heidelberglaan 100, 3584 CX Utrecht, The Netherlands; 7 Department of Radiation Oncology, University Medical Center Utrecht, Heidelberglaan 100, 3584 CX Utrecht, The Netherlands

**Keywords:** nasal vestibule, squamous cell carcinoma, sinonasal cancer, staging

## Abstract

**Simple Summary:**

Cancer of the nasal vestibule is thought to be rare. Multiple staging systems exist for the staging of these tumors, which can lead to variability and, therefore, the poor reliability of data. This retrospective study aimed to evaluate these staging systems. One hundred and forty-eight patients with carcinoma of the nasal vestibule were included and re-staged per the available staging systems. Stage distribution varied widely between the four evaluated staging systems. The classification per Bussu et al. had the most balanced allocation of patients amongst the stages. The widespread adoption of a single system and the introduction of a designated topography code for this disease could lead to greater uniformity in data reporting and an improved understanding of the incidence and disease outcome. Further analysis of survival data is needed to assess which classification system is the best suited for nasal vestibule carcinoma.

**Abstract:**

Squamous cell carcinoma of the nasal vestibule is reported to account for less than one percent of all head and neck malignancies. It lacks a designated WHO ICD-O topography code, and multiple systems are available for the staging of this disease, which results in unwanted variability and the subsequent poor reliability of data. The aim of this study was to evaluate the currently available staging systems for cancer of the nasal vestibule, including the recently introduced classification by Bussu et al., which built on Wang’s original concept but with clearer anatomical cutoffs. Different staging systems for cancer of the nasal vestibule (UICC nasal cavity, UICC skin cancer of the head and neck, Wang and Bussu et al.) were evaluated via a retrospective analysis of 148 patients. The staging system, per Bussu et al., had the most balanced allocation of patients among the stages. When using the Wang classification as a reference, stage migration occurred less frequently with the Bussu classification. The widespread adoption of a single staging system, as well as the introduction of a designated topography code for cancer of the nasal vestibule, could lead to more uniformity in data reporting and improve an understanding of the incidence and disease outcome. The newly proposed carcinoma of the nasal vestibule classification by Bussu et al. has the potential to improve the staging and allocation among stages. Further analysis of survival data is needed to assess which classification system is best suited for nasal vestibule carcinoma.

## 1. Introduction

Within the overarching spectrum of sinonasal malignancies, cancer of the nasal vestibule (CNV) constitutes a distinct entity. The anatomical site, located at the intersection of external and internal body surfaces, forms a transition area from the keratinized stratified squamous epithelium of the facial skin to the pseudostratified ciliated columnar (respiratory) epithelium that lines most of the nasal cavity proper. Malignant tumors that arise in this area, the vast majority of which are squamous cell carcinoma (SCC), are deemed rare and comprise less than one percent of all head and neck cancer [1]. Because of their location, the tumors are generally noticeable at an early-stage, resulting in early diagnosis [2]. Both surgery and primary radiotherapy (RT) provide excellent oncological outcomes in the case of early stage disease, but surgical treatment can result in facial disfigurement and the subsequent need for reconstruction or epithesis [3,4,5,6,7,8,9,10]. Brachytherapy, also known as interventional radiotherapy, has recently become the preferred treatment modality for early-stage CNV since it provides superior survival and aesthetic and functional outcomes compared to surgery or external beam radiotherapy [11,12,13,14,15,16,17]. Meanwhile, surgery followed by RT remains the cornerstone of treatment for advanced CNV.

Several separate staging systems are available at present, although the frequency of their use in clinical practice varies. These include the Union for International Cancer Control (UICC) TNM classification (8th edition) for tumors of the nasal cavity and paranasal sinuses, in which the nasal vestibule is considered part of the nasal cavity (WHO ICD-O code 30.0), which, in turn, shares T-classification criteria with the ethmoid sinus (code 30.1); the Wang classification, which was developed specifically for the nasal vestibule; and the UICC TNM-classification (8th edition) for non-melanoma skin cancer of the head and neck [18,19]. Each of the aforementioned options has its advantages and disadvantages and does not touch on all relevant aspects of CNV. As a result, while multiple studies have advocated the Wang classification by virtue of its relative simplicity and superior prognostic value compared to UICC classifications, a true consensus is lacking [11,16,20,21,22]. Such variety and lack of uniformity of the data limit our understanding of CNV and hinder reaching a broad consensus regarding treatment. Moreover, since the nasal vestibule has not been assigned a unique topography code, misclassification may have led to an underestimation of incidence.

As a response to the apparent shortcomings of the current staging systems, Bussu et al. proposed a novel T-classification for CNV structured around the premise of the nasal vestibule as a separate site with a special focus on the area’s unique anatomy and the disease’s peculiar pattern of spread [23,24]. This multicenter retrospective cohort study aimed to compare the most commonly used T-staging methods with regard to stage distribution and migration.

## 2. Materials and Methods

In this retrospective multicenter cohort study, all consecutive patients who had been treated for primary CNV across three tertiary referral centers for head and neck oncology between August 2004 and December 2022 were included. Parts of this cohort have been described in previous publications [4,16,17,25,26]. All patients underwent a clinical evaluation, including the histopathological confirmation of diagnosis and subsequent imaging studies, which typically consisted of either magnetic resonance imaging (MRI) or computed tomography (CT) of the head and neck, chest X-ray/CT, and neck ultrasound with or without the fine-needle aspiration cytology of suspicious lymph nodes. Relevant data were extracted from patient health records. The age at the time of diagnosis was determined on the date of histopathological confirmation. Through reviewing the clinical data and imaging studies, all tumors were restaged per the 8th edition of the UICC TNM classification for tumors of the nasal cavity and paranasal sinuses, as well as non-melanoma skin cancer of the head and neck. Additionally, tumors were restaged in accordance with the Wang and Bussu et al. staging systems (Table 1). The Wang classification was used as the baseline when evaluating the stage migration between systems. Tumors that migrated from Wang T3 to either T3 or T4a in other classifications were deemed not to have migrated since the Wang classification consisted of only three separate stages. All patients gave informed consent for the use of their medical photographs.

The study was approved by the medical ethics committee (22-859, 20 September 2022). For this type of study, formal consent was not required. Participating centers complied with local medical ethics committee requirements. 

## 3. Results

A total of 148 patients who were treated for a CNV across three European tertiary head and neck referral centers were identified and included. The clinical and treatment characteristics of these patients are displayed in Table 2.

Pre-treatment imaging protocols varied over time and between participating centers. Out of 148 patients, 90 (60.8%) and 106 (71.6%) received a CT scan or MRI of the head and neck, respectively. Of these, 55 patients (37.2%) received both. For further staging purposes, the majority of patients received either a neck ultrasound (84.5%) and/or chest imaging (76.4%), whilst 28 patients (18.9%) received a positron emission tomography (PET). Imaging was not performed in two patients (1.4%) who refused further treatment.

**Figure 1:**
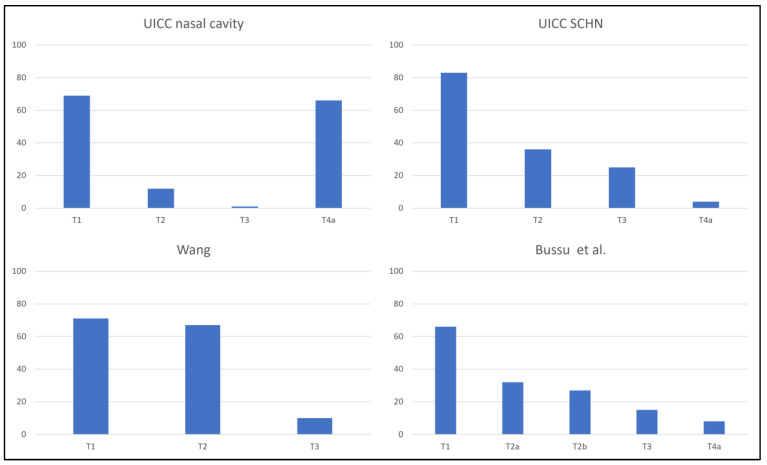
Distribution of patients (n) amongst the different stages per staging system. UICC = Union for International Cancer Control; SCHN = skin cancer of the head and neck.

**Figure 2:**
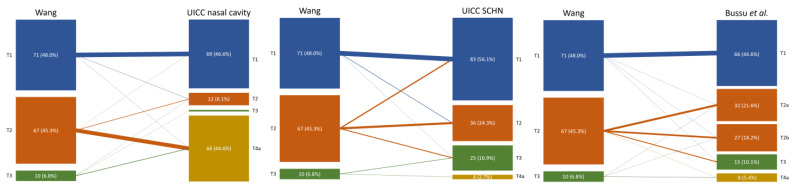
Stage migration from the Wang classification to the other staging systems. The thickness of the interconnecting lines was scaled to represent the corresponding percentage of patients. UICC = Union for International Cancer Control; SCHN = skin cancer of the head and neck.

When staged according to the 8th edition of the UICC TNM classification for sinonasal malignancies, almost half of the patients (44.6%) had a T4a tumor, whilst only one patient (0.7%) had a T3 disease (Figure 1). The percentage of T3/T4a tumors was lower when staged per the 8th edition TNM-classification for skin cancer of the head and neck, Wang, or Bussu et al.: 29 (19.6%), ten (6.8%), and 23 (15.5%), respectively. Stage migration occurred frequently but differed between systems (Table 3, Figure 2). Using the Wang classification as a baseline, tumors were often upstaged after restaging per other systems. Stage migration was least common when restaging, per Bussu et al. Nine (6.1%) patients were presented with cervical lymph node metastases. Of these, one patient had a T1 tumor (Wang), while four and four patients presented with T2 or T3 disease (Wang). In all these patients, lymph node metastases were found in the neck, whilst none were found in the nasolabial folds. None of the patients had distant metastases at the initial presentation. The mean tumor diameter, when recorded (n = 98), was 18.0 mm (SD = 1.2). In this cohort, 113 (95.6%) patients received brachytherapy, 15 (10.1%) received EBRT, and three (2.0%) received a combination of both. Fourteen (9.5%) patients underwent surgery, either with or without adjuvant EBRT, and one patient (0.7%) received chemoradiotherapy (cisplatin/etoposide + EBRT) for a tumor with invasion of the maxilla.

## 4. Discussion

This multicenter retrospective cohort study described a total of 148 patients who were treated for primary CNV with the aim of evaluating and comparing the available staging systems.

A disease classification ideally allowed for the stratification of patients based on a set of clear-cut criteria. The distribution amongst its categories should be such that a physician could both decide on which treatment modality to employ as well as estimate an individual patient’s prognosis based on their disease stage.

The distribution of patients varied widely between the different staging systems. Nearly half of all patients required upstaging (compared to Wang) when employing the UICC 8th edition of the TNM classification for tumors of the nasal cavity, which in nearly all cases was due to skin involvement. Meanwhile, only two patients were staged at T3, resulting in an unbalanced allocation of patients among the stages. By contrast, using the UICC 8th edition TNM classification for skin cancer of the head and neck led to the downstaging of 17 T2 tumors (Wang) to T1, showing that tumors did not need to be large in order to invade adjacent structures. Even the utilization of the system per Bussu et al., which built on Wang’s classification, led to upstaging in nearly one-quarter of patients but seemed to produce a more balanced distribution of patients compared to the other staging systems. The skewed distribution and the prevalence of stage migration between the systems only further emphasized the fundamental differences between classifications which caused a lack of uniformity in the data. In addition, these staging systems also posed a number of practical issues.

### Pitfalls and Benefits of Available Staging Systems

In the 8th edition of the TNM classification, malignancies of the nasal vestibule were assigned the exact same ICD-10 topography code as tumors of the nasal cavity proper (C30.0). By contrast, the maxillary sinus (C31.0), ethmoid sinus (C31.1), and even the frontal (C31.2) and sphenoid sinuses (C31.3) received designated identifiers. As such, CNV could not be clearly distinguished as a separate entity and was adequately entered in major registries such as the National Cancer Database (NCDB) and the Surveillance, Epidemiology, and End Results (SEER) program, which were crucial for the accurate estimation of disease incidence. These authors advocated for the introduction of a novel topography code, distinguishing the nasal vestibule from the nasal cavity proper and the paranasal sinuses. Similarly, the TNM classification for tumors of the nasal cavity and paranasal sinuses was evidently not designed for the nasal vestibule and posed an array of challenges when used in clinical practice. Firstly, its definition of what exactly comprised the nasal vestibule is unclear. Consequently, differentiating between T1 and T2 tumors has become unnecessarily confusing. This distinction was made based on the number of subsites involved. Paradoxically, the vestibule also encompassed the anterior septum, which itself was considered a separate subsite. Moreover, tumors could be staged in T1 regardless of the invasion of bony structures: a feature that is common in tumors of the nasal cavity proper or paranasal sinuses but is typically observed only in locally advanced CNV. Another frequently encountered issue is skin invasion, which immediately requires a tumor to be staged at T4a. This feature is commonplace because of the invasion of either the columella or the nasal alae, as illustrated by the percentage of T4a tumors in our cohort. Meanwhile, the elements necessary for a tumor to be staged T3 are rare and would require a significantly vaster extension of the primary tumor. Nevertheless, the structure and uniformity of the UICC TNM classification provided as a whole are unprecedented. The integration of a dedicated system for CNV into this broader framework would, therefore, be a clear improvement.

The UICC TNM classification for skin cancer of the head and neck is used more sparingly for the staging of CNV. It distinguishes between different T-stages on the basis of tumor dimensions and invasion depth. Tumors with an invasion depth of >6 mm or invasion beyond the subcutaneous fat were staged T3. In contrast to the external skin, the layer of subcutaneous tissue in the nasal vestibule was relatively thin and, in some areas, barely present at all. The depth of invasion, in particular of the cartilage and bone, and increased tumor diameter have been shown to be poor prognostic indicators (14). However, the use of this classification can lead to the unnecessary upstaging of relatively small tumors that may otherwise be easy to treat.

The Wang classification, first proposed in 1976, is the only presently available staging system that is tailored specifically to the nasal vestibule and is commonly used in specialized referral centers. Furthermore, its prognostic value has been proven to be superior to the aforementioned staging systems (14, 16). It is centered around the unique anatomy of the vestibule, and upstaging occurs as tumors invade adjacent structures such as the upper lip or turbinates. Yet, its vague phrasing allows for different interpretations and unnecessary variation in the clinical data. It is left entirely up to the individual physician to determine whether a tumor is deemed superficial or massive without providing concrete instructions. In the era of high-resolution imaging, this classification falls short in definitions. Moreover, because Wang’s classification consists of only three stages, its ability to discriminate and stratify is impaired. As a result, both tumors that are difficult to treat due to invasion of adjacent structures and tumors that are relatively bulky but superficial fall under the same category (T3).

**Figure 3:**
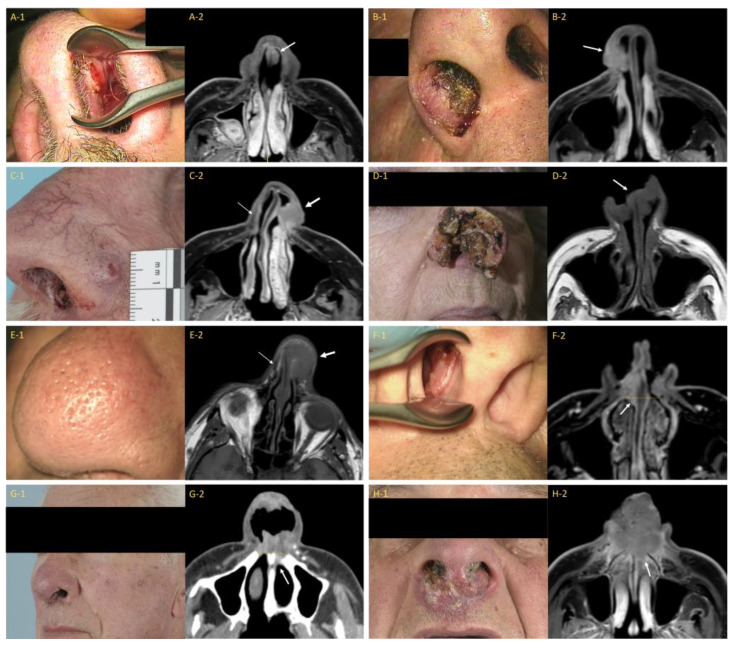
Clinical photographs and corresponding imaging studies of patients with a carcinoma of the nasal vestibule for each stage of the Bussu et al. classification. (**A-1**) = Patient with a left-sided T1 carcinoma of the nasal vestibule (CNV); (**A-2**) = Corresponding axial postcontrast T1-weighted MR image with fat saturation (T1 MR Gd FS) showing subtle soft tissue thickening on the left side of the nasal septum (white arrow); (**B-1**) = Patient with a right-sided T2a CNV (caudal ala); (**B-2**) = Corresponding axial T1 MR Gd FS image displaying the tumor invading the cutis (white arrow); (**C-1**) = Patient with a left-sided T2b CNV with extension through the alar cartilage to the external skin of the nose; (**C-2**) = Corresponding axial T1 MR Gd FS image showing cartilage invasion and an irregular skin contour (thick white arrow). Compare to the contralateral side where the normal (low signal intensity) alar cartilage is visible (thin white arrow); (**D-1**) = Patient with a T2b CNV with extensive tissue destruction; (**D-2**) = Corresponding axial T1 MR Gd FS image showing the (bilateral) tumor (white arrow); (**E-1**) = Patient with a left-sided T2b CNV with peau d’orange suggesting the invasion of the (sub)cutis; (**E-2**) = Corresponding axial T1 MR Gd FS image. Note: absence of the alar cartilage (thick white arrow). Normal (low signal intensity) alar cartilage is present on the contralateral side (thin white arrow); (**F-1**) = Patient with a right-sided T3 CNV; (**F-2**) = Corresponding axial T1 MR Gd FS image shows tumor extension (white arrow) beyond the plane of the pyriform aperture (yellow line); (**G-1**) = Patient with a left-sided T3 CNV; (**G-2**) = Corresponding axial postcontrast CT image showing extension (white arrow) beyond the plane of the pyriform aperture (yellow line); (**H-1**) = Patient with a T4a CNV; (**H-2**) = Corresponding axial T1 MR Gd FS image displaying extension of the tumor into the anterior maxilla (white arrow).

This classification was originally proposed by Bussu et al., who built on Wang’s system but incorporated clearly delineated anatomical landmarks to differentiate between the stages [23]. It defines the nasal vestibule as the area extending from the plane tangential to the pyriform aperture to the nostrils. Tumors are upstaged based on the invasion of the skin of the face or external nose (T2a), cartilage invasion (T2b), posterior extension beyond the pyriform aperture (T3), bone invasion (T4a), and the invasion of adjacent vital structures such as the orbit or anterior cranial base (T4b). An overview of the clinical images and corresponding imaging studies is provided in Figure 3. This classification aims to be both easy to use in clinical practice as well as providing a better indicator of prognosis compared to existing classifications. The prognostic value of this classification still requires additional validation.

## 5. Conclusions

Squamous cell carcinoma of the nasal vestibule is a malignancy without a dedicated UICC TNM-staging system. The four staging systems (UICC nasal cavity, UICC SCHN, Wang and Bussu et al.) evaluated in this study employ different methods of categorizing CNV. Stage migration occurred frequently when restaging patients, thereby underlining the differences between them. The classification system proposed by Bussu et al. seems to provide a more balanced allocation of patients among the stages, but its prognostic value requires additional analysis.

## Figures and Tables

**Table 1:** T-staging systems for cancer of the nasal vestibule. UICC = Union for International Cancer Control. n.a. = not applicable. * Deep invasion is defined as invasion beyond the subcutaneous fat or >6 mm (as measured from the granular layer of the adjacent normal epidermis to the base of the tumor), perineural invasion for T3 classification is defined as clinical or radiographic involvement of named nerves without a foramen or skull base invasion or transgression.

	UICC 8th ed. Nasal Cavity [18]	UICC 8th ed. SCHN [18]	Wang [19]	Bussu et al. [23]
T1	Tumor restricted to any one subsite, with or without bony invasion.	Tumor 2 cm or less in greatest dimension.	The lesion is limited to the nasal vestibule and is relatively superficial, involving one or more subsites within.	Tumor limited to the internal lining of the nasal vestibule (skin and/or mucosa).
T2a	Tumor invading two subsites in a single region or extending to involve an adjacent region within the nasoethmoidal complex, with or without bony invasion.	Tumor > 2 cm and ≤4 cm in greatest dimension.	The lesion has extended from the nasal vestibule to adjacent structures, such as the upper nasal septum, upper lip, philtrum, skin of the nose and/or nasolabial fold, but is not fixed to the underlying bone.	Tumor invading superficial structures (cutis, subcutis) beyond the nasal cavity, in particular the external surface of the nose, the nasolabial fold, philtrum, or upper lip, without invasion of cartilage, bone, or structures beyond the plane of the pyriform aperture.
T2b	n.a.	n.a.	n.a.	Tumor invading cartilage (quadrangular, triangular, alar), without invasion of bony structures or structures beyond the plane of the pyriform aperture.
T3	Tumor extends to invade the medial wall or floor of the orbit, maxillary sinus, palate, or cribriform plate.	Tumor > 4 cm in greatest dimension or minor bone erosion or peri- neural invasion or deep invasion. *	The lesion has become massive with extension to the hard palate, buccogingival sulcus, including a large portion of the upper lip, upper nasal septum, turbinate and/or adjacent paranasal sinuses, fixed with deep muscle and bone involvement.	Tumor extends internally beyond the plane of the pyriform aperture, with or without cartilage invasion, but without bone invasion.
T4a	Moderately advanced local disease. Tumor invades any of the following: anterior orbital contents, skin of nose or cheek, minimal extension to anterior cranial fossa, pterygoid plates, sphenoid or frontal sinuses.	Tumor with gross cortical bone/marrow invasion.	n.a.	Tumor invades bony structures (e.g., hard palate, nasal bones, frontal process of the maxilla, ethmoid, or orbit).
T4b	Very advanced local disease. Tumor invades any of the following: orbital apex, dura, brain, middle cranial fossa, cranial nerves other than (V2), nasopharynx, or clivus.	Tumor with skull base or axial skeleton invasion including foraminal involvement and/or vertebral foramen involvement in the epidural space.	n.a.	Tumor invades any of the following: orbital apex, dura, brain, middle cranial fossa, cranial nerves other than (V2), nasopharynx, or clivus.

**Table 2:** Patient and treatment characteristics of patients with cancer of the nasal vestibule. UICC = Union for International Cancer Control; SCHN = skin cancer of the head and neck; EBRT = external beam radiotherapy.

Sex	N	%
Total	148	100
Male	95	64.2
Female	53	35.8
Age	N	IQR
Median age in years	70	63–77
Imaging studies	N	%
MRI-head/neck	106	71.6
CT-head/neck	90	60.8
Neck ultrasound	125	84.5
Chest X-ray/CT	113	76.4
PET/CT	28	18.9
None	2	1.4
T-stage at diagnosis	N	%
UICC nasal cavity		
T1	69	46.6
T2	12	8.1
T3	1	0.7
T4a	66	44.6
T4b	0	0
UICC SCHN		
T1	83	56.1
T2	36	24.3
T3	25	16.9
T4a	4	2.7
T4b	0	0
Wang		
T1	71	48.0
T2	67	45.3
T3	10	6.8
Bussu et al.		
T1	66	44.6
T2a	32	21.6
T2b	27	18.2
T3	15	10.1
T4a	8	5.4
T4b	0	0
N-stage at diagnosis	N	%
N0	139	93.9
N+	9	6.1
Tumor diameter	N	%
<15 mm	46	31.1
≥15 mm	52	35.1
Unknown	50	33.8
Primary tumor treatment modality	N	%
Brachytherapy	113	76.4
EBRT	15	10,1
Brachytherapy + EBRT	3	2.0
Surgery	8	5.4
Surgery + EBRT	6	4.1
Chemoradiotherapy	1	0.7
Best supportive care	2	1.4
Neck treatment modality	N	%
EBRT	2	1.4
Neck dissection	7	4.7
Neck dissection + EBRT	1	0.7

**Table 3:** Migration of T-stages. The Wang classification served as the baseline for determining the number of patients who were up-staged or down-staged. UICC = Union for International Cancer Control; SCHN = skin cancer of the head and neck.

	UICC Nasal Cavity		UICC SCHN		Bussu et al.	
Stage Migration	N	%	N	%	N	%
Upstaged	61	41.2	26	17.6	19	12.8
Downstaged	3	2.0	18	12.2	3	2.0
No stage migration	84	56.8	104	70.3	126	85.1

## Data Availability

The data presented in this study are available on request from the corresponding author. The data are not publicly available due to privacy-related restrictions.
